# A comparison of three approaches for the discovery of novel tripartite attachment complex proteins in *Trypanosoma brucei*

**DOI:** 10.1371/journal.pntd.0008568

**Published:** 2020-09-16

**Authors:** Hélène Clémentine Margareta Baudouin, Laura Pfeiffer, Torsten Ochsenreiter

**Affiliations:** 1 Institute of Cell Biology, University of Bern, Bern, Switzerland; 2 Graduate School for Cellular and Biomedical Sciences, University of Bern, Bern, Switzerland; Hunter College, CUNY, UNITED STATES

## Abstract

*Trypanosoma brucei* is a single celled eukaryotic parasite and the causative agent of human African trypanosomiasis and nagana in cattle. Aside from its medical relevance, *T*. *brucei* has also been key to the discovery of several general biological principles including GPI-anchoring, RNA-editing and trans-splicing. The parasite contains a single mitochondrion with a singular genome. Recent studies have identified several molecular components of the mitochondrial genome segregation machinery (tripartite attachment complex, TAC), which connects the basal body of the flagellum to the mitochondrial DNA of *T*. *brucei*. The TAC component in closest proximity to the mitochondrial DNA is TAC102. Here we apply and compare three different approaches (proximity labelling, immunoprecipitation and yeast two-hybrid) to identify novel interactors of TAC102 and subsequently verify their localisation. Furthermore, we establish the direct interaction of TAC102 and p166 in the unilateral filaments of the TAC.

## Author summary

*Trypanosoma brucei* belongs to a group of organisms that exist as human, animal and plant parasites. *T*. *brucei* (a human and animal parasite) has been developed as a model system to study basic biological as well as disease related questions in this group of organisms. We study how the parasite duplicates and divides its mitochondrial genome, an essential component of its energy generating machinery. The structure involved in dividing the mitochondrial genome into the daughter cells during cell division is called the tripartite attachment complex (TAC). The TAC is likely a unique structure not present in the host and thus might provide a new avenue for drug development. In this manuscript, we compare different techniques that allow the identification of novel components of this structure and verify the localisation of some of them. Furthermore, we also establish the interaction of two previously identified protein components.

## Introduction

*Trypanosoma brucei*, a protist parasite, is the causative agent of human African trypanosomiasis and nagana in cattle [1,2]. This single celled eukaryote belongs to group Kinetoplastea, which is characterised by the presence of a singular yet complex mitochondrial genome consisting of a DNA network referred to as kinetoplast DNA or kDNA [3–5]. The kDNA in *T*. *brucei* consists of 25 almost identical maxicircles (23 kbp) that are linked to thousands of 1 kbp size minicircles, which are in turn catenated to each other. The maxicircles encode for 18 protein genes that are involved in oxidative phosphorylation and the mitochondrial ribosome as well as two ribosomal RNAs. Most (12) of the maxicircle protein coding genes are pseudogenes and require posttranscriptional insertion and/or deletion of uridine residues in order to be translatable [6,7]. This process, called RNA editing, requires a 34S ribonucleotide protein complex consisting of more than 20 different proteins as well as small, 50–70 nucleotides, guide RNAs (gRNAs) that define the editing pattern [8–10]. The gRNAs are encoded on the minicircles of the network. A recent study showed that the *T*. *brucei* mitochondrial genome harbours about 400 different minicircle sequences in the network coding for 1300 gRNA genes [11].

The complexity of replicating the kDNA rivals its structure, and although more than 30 components have been characterised, the compendium of the kDNA replication machinery is far from complete [12,13]. Since the parasite contains a single mitochondrion with one genome per cell, proper segregation of these two entities is critical for cell proliferation. In the bloodstream form parasite, the mitochondrion grows in two regions anterior and posterior of the nucleus building a network that is pruned prior to separation in the two daughter cells [14]. The segregation of the replicated kDNA network is carried out by the tripartite attachment complex (TAC) that is composed of three parts: (i) the exclusion zone filaments, connecting the basal body of the flagellum to the outer mitochondrial membrane; (ii) the two differentiated mitochondrial membranes and (iii) the unilateral filaments connecting the inner mitochondrial membrane to the kDNA [15]. The current model of the TAC contains 13 components [16]. Four of which (p197, BBA4, Mab22 and TAC65) are localised to the exclusion zone filaments [17–19]. Six components are in the differentiated mitochondrial membranes, four in the outer mitochondrial membrane (TAC60, TAC42, TAC40 and pATOM36, [19–21]), and two components are likely associated with the inner mitochondrial membrane (p166, AEP1, [22,23]). TAC102 is a protein of the unilateral filaments and the most proximal component to the kDNA [24,25]. A number of additional components that display multiple localisations including in the TAC, like the E2 subunit of the α-ketoglutarate dehydrogenase and the tubulin-binding cofactor C protein, have been identified [26,27]. The assembly of the TAC occurs *de novo*, from the base of the flagellum towards the kDNA, in a hierarchical manner, such that kDNA proximal components depend on the proper assembly of the kDNA distal components [16,28]. As TAC102 is the protein most closely apposed to the kDNA, and therefore one of the last to be added to the replicating complex, it was selected to identify novel proteins that connect the TAC to the kDNA. We used three different approaches: (i) proximity-dependent biotin identification (BioID), (ii) immunoprecipitation with a monoclonal TAC102 antibody and (iii) yeast two-hybrid. BioID was first used for the identification of protein-protein interactions in mammalian cells [29]. It involves the expression of a protein of interest fused to a modified version of a bacterial biotin ligase (BirA*, [30]), in order to identify protein partners in close proximity. The original enzyme, BirA, adds an ATP to biotin in order to form biotinoyl-5’-AMP, which is highly reactive [31]. This intermediate is then retained in the active site of BirA until the enzyme finds a lysine in the target protein. The biotinoyl-5’-AMP reacts with the lysine and the protein is finally biotinylated. BirA* has a lower affinity for the intermediate biotinoyl-5’-AMP. Once it is produced, it is released from the active site of BirA*. This results to biotinylation of all proteins in a radius of 20 nm around BirA* [32]. The biotinylated proteins can then be purified using streptavidin beads and are identified by mass spectrometry. The BioID approach was previously applied to identify interacting partners of the hook complex protein TbMORN1, a key component of the cytoskeleton-associated structure close to the flagellar pocket of *T*. *brucei* [33]. Immunoprecipitation has widely been used to characterise protein-protein interactions in *T*. *brucei*. However, in most studies the protein of interest has been tagged, which can potentially lead to a change in protein expression levels as well as interference of the tag with the protein’s function. In this study, we used a monoclonal antibody raised against a region in the C-terminus of TAC102. This antibody was previously shown to be highly specific for TAC102 in fixed cells and native/denatured protein extracts [24,28]. The third approach we applied was a yeast two-hybrid screen that relies on the reconstitution of a functional transcription factor when two peptides of interest interact [34]. One of the advantages of the yeast two-hybrid screen is its ability to identify interaction domains through the expression of parts of the bait/prey proteins. However, for the same reason false positive/negative interactions are possible since the peptides might fold differently than the entire protein. Yeast two-hybrid screens have successfully been used in *T*. *bruce*i, albeit much less frequently than immunoprecipitation approaches. Two examples reporting protein-protein interactions in the parasite are the characterisation of a SUMOylation factor interacting with the transcription machinery regulating VSG expression and the description of mitochondrial protein-protein interactions in the RNA editing accessory complex MRB1 [35,36].

Here, we compare the three approaches for the discovery of novel interactors of TAC102, an essential component of the mitochondrial DNA segregation machinery in *T*. *brucei*. We identify nine proteins to be enriched in both the BioID and the immunoprecipitation approach, three of which we localise to the TAC/kDNA region. The yeast two hybrid and the immunoprecipitation approach identified p166, a previously described TAC component, as the main interactor of TAC102.

## Results

### Myc-BirA*-TAC102 is active and colocalises with the endogenous TAC102

**Fig 1:**
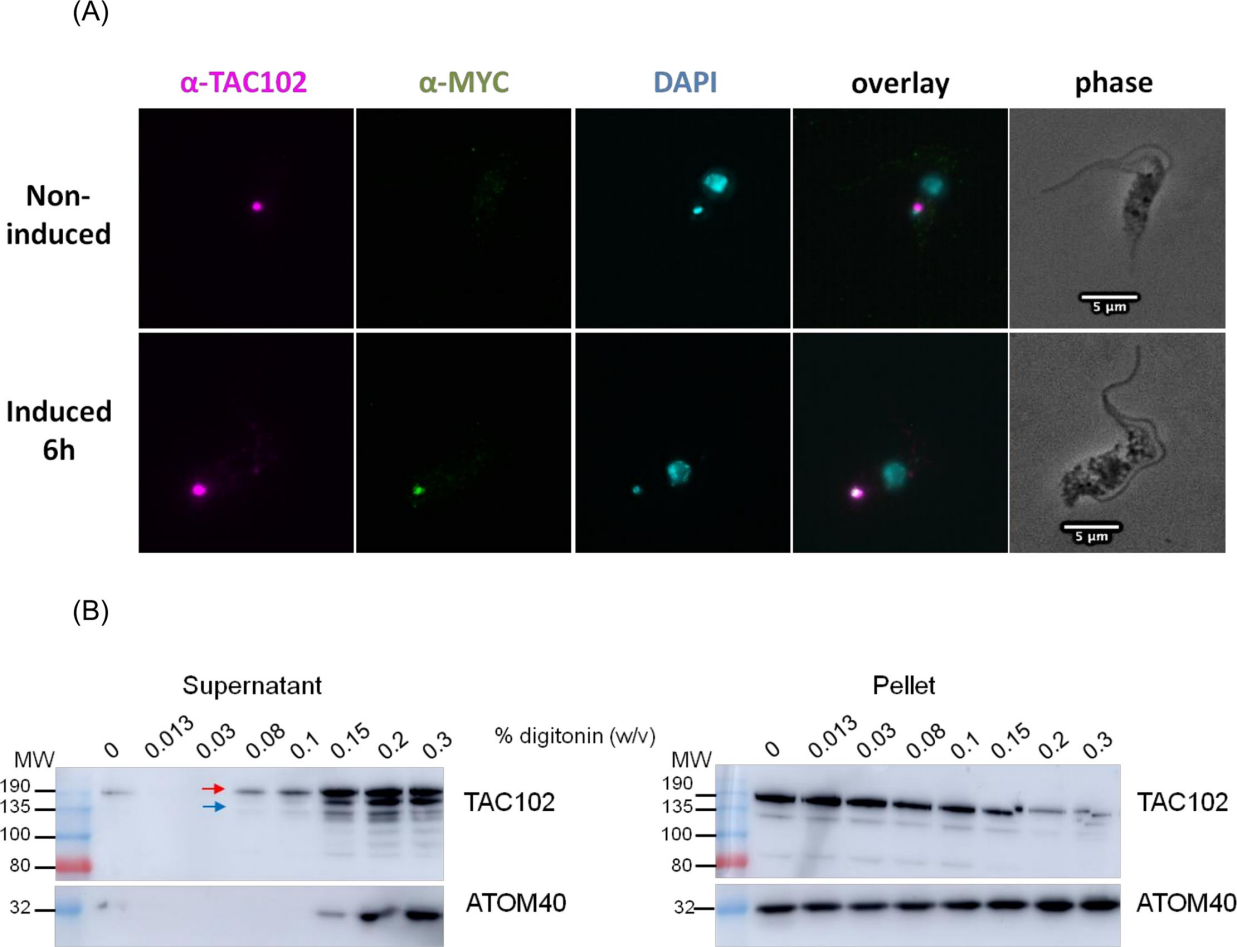
Characterisation of the Myc-BirA*-TAC102 cell line. (A) Immunofluorescence microscopy pictures of Myc-BirA*-TAC102 PCF cells. A monoclonal antibody was used to visualise TAC102 (magenta). An anti-Myc antibody was used to visualise Myc-BirA*-TAC102 (green). DAPI stained nuclei and kDNA (cyan). Around 100 cells were observed before choosing the example pictures. (B) Western blot analysis of digitonin fractionated cell extracts from the Myc-BirA*-TAC102 cell line (PCF) after 24 hours of induction with tetracycline. Myc-BirA*-TAC102 (red arrow) and the endogenous TAC102 (blue arrow) were detected using the monoclonal anti-TAC102 antibody. Molecular weights (MW) are in kDa. As control, we probed for the mitochondrial membrane protein ATOM40. The digitonin fractionation was done twice and both experiments showed the same results.

The Myc-BirA*-TAC102 fusion construct was integrated into the ribosomal array in PCF cells allowing for inducible expression through the addition of tetracycline. After six hours of induction, we visualised the expression of the Myc-BirA*-TAC102 fusion protein by immunofluorescence microscopy (Fig 1A) and found it to be colocalised with the endogenous TAC102 (Tb927.7.2390) in the posterior region of the mitochondrion, close to the kDNA. In order to test if the fusion protein associates with the TAC structure similarly as we previously showed for the endogenous TAC102 [24], we evaluated the solubility using increasing amounts of the non-ionic detergent digitonin during a biochemical fractionation. For this analysis, the Myc-BirA*-TAC102 fusion protein was expressed for 24 hours. The supernatants from the digitonin fractions were separated on a SDS-PAGE for western blot analysis (Fig 1B) demonstrating that the majority of Myc-BirA*-TAC102 was solubilised at a similar concentration of digitonin (0.2%) as previously described for the endogenous TAC102 [24]. Aside from the signal for the Myc-BirA*-TAC102 fusion protein (about 135 kDa) and the weaker signal for TAC102 (102 kDa), we also detected several additional signals (see discussion).

### Myc-BirA*-TAC102 is able to biotinylate proteins

**Fig 2:**
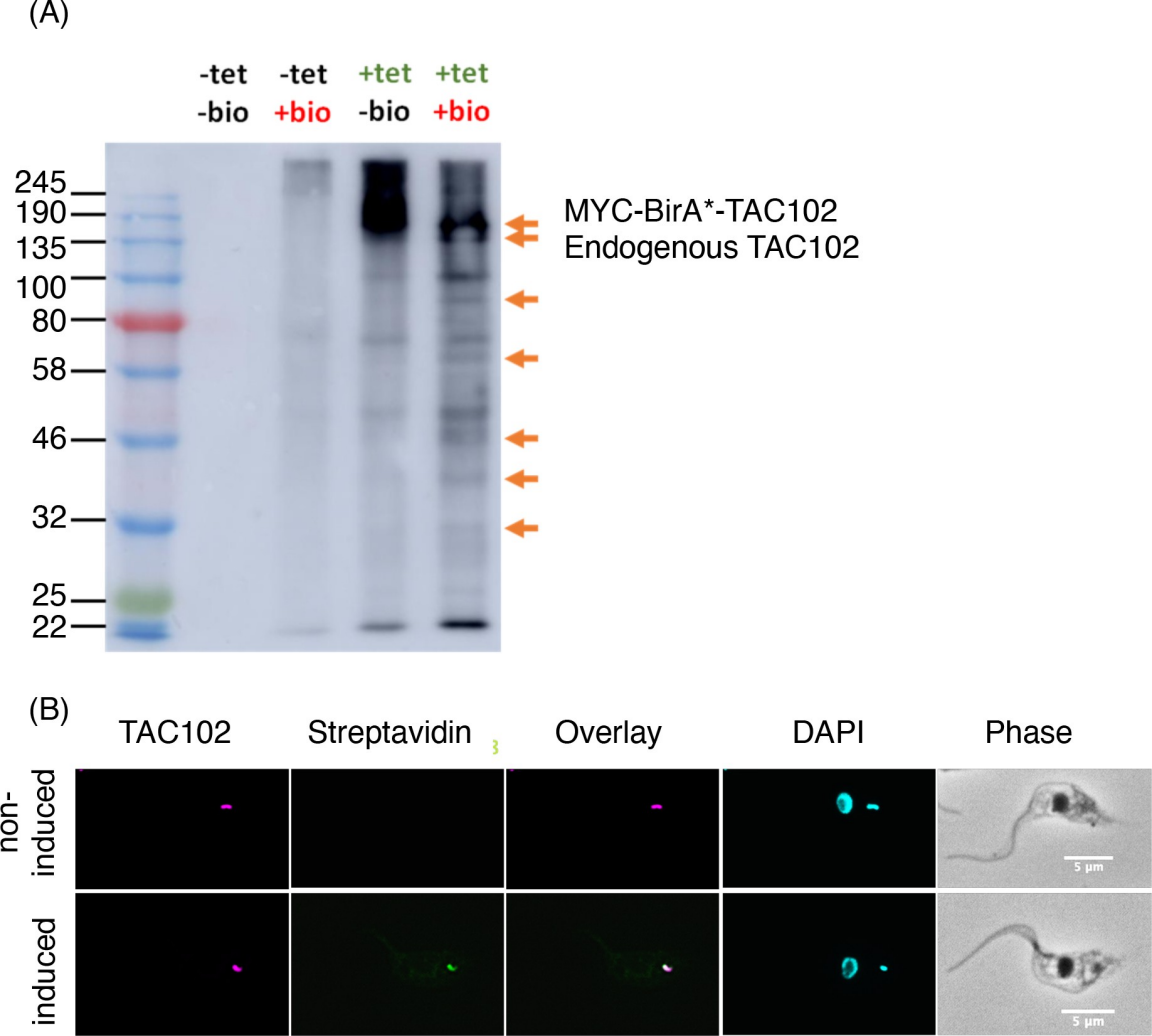
Biotinylation by Myc-BirA*-TAC102 in PCF cells. (*A*) Western blot analysis of biotinylated proteins from the Myc-BirA*-TAC102 cell line (PCF) with (“+ tet”) or without (“- tet”) induction with tetracycline overnight and with (“+ bio”) or without (“- bio”) addition of biotin in the medium. Left lane indicates protein size in kDa. Arrows indicate examples of proteins only detected in the condition “+ tet, + bio”. The two upper arrows indicate the expected size for Myc-BirA*-TAC102 and for the endogenous TAC102 protein. Data were obtained from one experiment. (B) Immunofluorescence microscopy pictures of Myc-BirA*-TAC102 cell line (PCF) with or without induction with tetracycline for five hours. A monoclonal antibody was used to visualise TAC102. Streptavidin-Alexa 488 recognised the biotinylated proteins. DAPI stained nuclei and kDNA. Around 100 cells were observed before choosing the example pictures.

**Fig 3:**
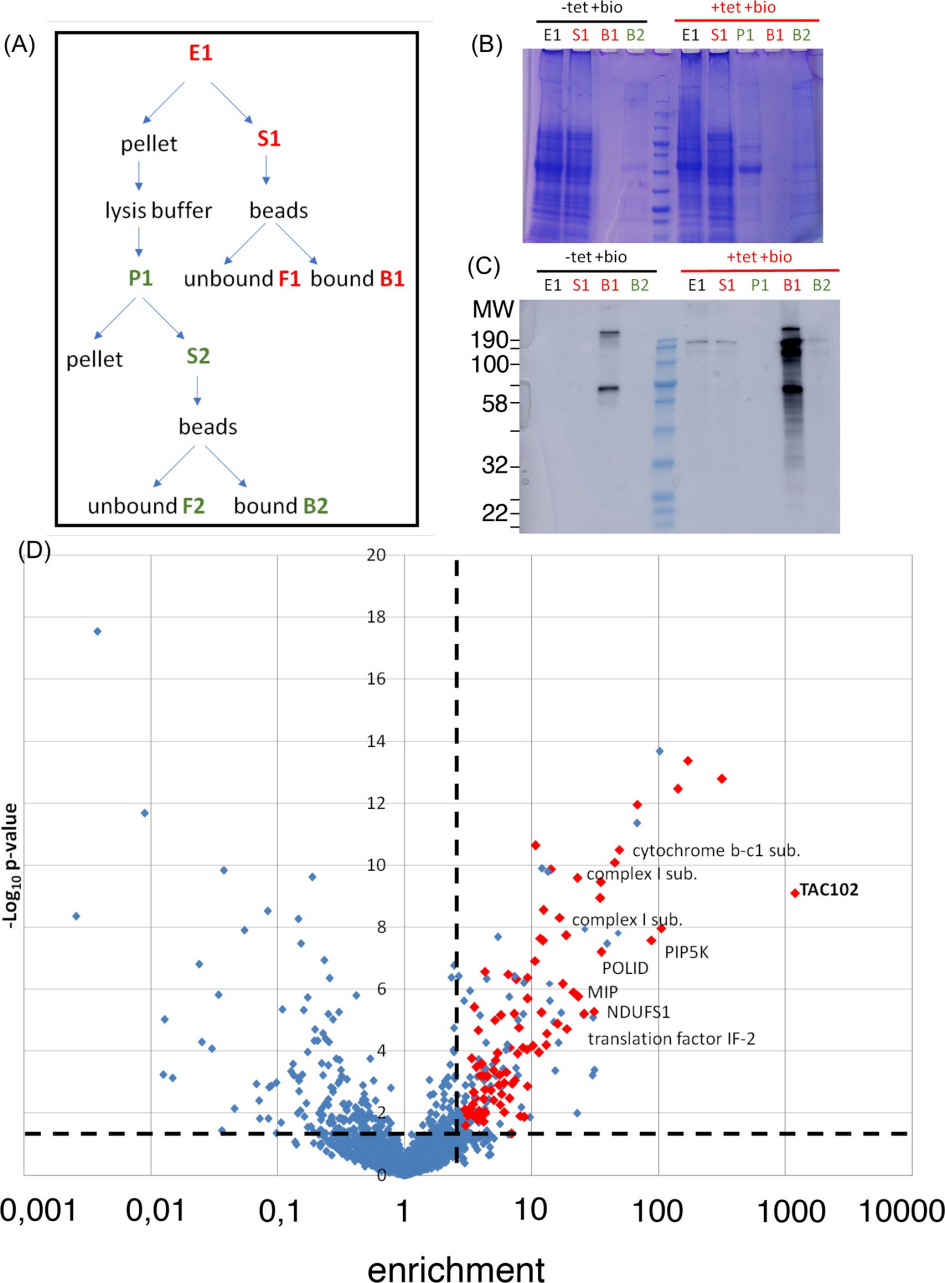
Purification of TAC102 binding partners and near neighbours using BioID. (*A*) Schematic of purification protocol. (*B*) Coomassie staining of the SDS-PAGE gel from BioID fractions. The experiment was done in four independent replicates and all showed the same results. (*C*) Western blot analysis of the BioID fractions. Streptavidin-HRP was used to detect the biotinylated proteins. Total cells extracts (E1), cleared supernatants (S1), first pellet (P1) and streptavidin bead bound (B1, B2) fractions were loaded on the gel. For E1, S1 and P1, the equivalence of 7.2x10^6^ cells was loaded. For B1 and B2, the equivalence of 137x10^6^ cells was loaded. MW: molecular weight. (*D*) TAC102 BioID enriched proteins (n = 4). The dashed lines represent the thresholds used for enrichment (> 3) and p-value (p < 0.05). Mitochondrial proteins identified with an enrichment > 3 (p < 0.05) are shown in red. IF: initiation factor; MIP: mitochondrial intermediate peptidase; NDUFS1: NADH-ubiquinone oxidoreductase complex I subunit; PIP5K: phosphatidylinositol-4-phosphate 5-kinase related; POLID: mitochondrial DNA polymerase I D; SSU: small subunit; sub.: subunit; tet: tetracycline.

In order to test the activity of the Myc-BirA*-TAC102 fusion protein inside the mitochondrial organelle, we induced its expression in procyclic form cells and then evaluated the biotinylation pattern of the total cell extract by western blot (Fig 2A). After induction of the fusion protein, we could detect an increase of biotinylated proteins when compared to the non-induced control. The major band visible between 135 and 190 kDa was likely the auto-biotinylated Myc-BirA*-TAC102 fusion protein (Fig 2A). The overall level of biotinylation further increased when exogenous biotin was added to a final concentration of 50 μM. Most biotinylated proteins were soluble in the lysis buffer (Fraction S1, Fig 3C). While they were readily detectable by western blot, the overall amount of biotinylated proteins was low as seen on Coomassie stained polyacrylamide gels (Fraction B1, Fig 3B). The localisation of the biotinylated proteins was analysed by epifluorescence microscopy using a streptavidin-conjugated fluorophore (Alexa Fluor 488, Fig 2B). For this, the cells were first incubated with biotin for 24 hours before adding tetracycline to induce expression of Myc-BirA*-TAC102 for five hours. The streptavidin-conjugated antibody recognised proteins almost exclusively around the kDNA disc, colocalising with the signal for TAC102 (Fig 2B). Thus, consistent with the localisation of the fusion protein, the biotinylated proteins were mostly in proximity to the kDNA.

TAC102 BioID was performed as described (Fig 3A). In brief, biotin was added to procyclic form cells 24 hours prior to the expression of Myc-BirA*-TAC102. After six hours of Myc-BirA*-TAC102 expression, the cells were lysed with detergent (0.5% Nonidet P-40) in a buffer containing protease inhibitors. After centrifugation, the soluble fraction was incubated with streptavidin-conjugated magnetic beads to bind and enrich the biotinylated peptides. After washing, the biotinylated peptides were released from the beads by boiling with Laemmli buffer. Protein identification was done by mass spectrometry. The enrichment of biotinylated proteins in cells induced for the expression of Myc-BirA*-TAC102 was compared to non induced cells.

### TAC102 BioID identifies mostly mitochondrial proteins

We analysed the biotinylation pattern induced by Myc-BirA*-TAC102 in four biological replicates. Enrichment in the condition containing tetracycline (“+ tet” condition) compared to the condition without tetracycline (“- tet” condition) was calculated for the detected proteins. A Student t-test was performed to determine the significance of the changes. Based on this analysis, TAC102 was the most enriched protein (Fig 3D). Overall, 77 proteins were enriched at least three fold (significance p ≤ 0.01) (S1 Table). Of these proteins, 47 were predicted to have a mitochondrial localisation. Sixteen of the 47 proteins with predicted mitochondrial localisation were annotated as hypothetical proteins and six of these were in the top ten most enriched proteins. Aside from the hypothetical proteins, we identified 11 translation factors, six RNA binding proteins, five DNA binding proteins, five components of the oxidative phosphorylation machinery, three mitochondrial import factors and two nuclear import/export proteins.

### Myc-BirA*-TAC102 seems to interact with MIP

**Fig 4:**
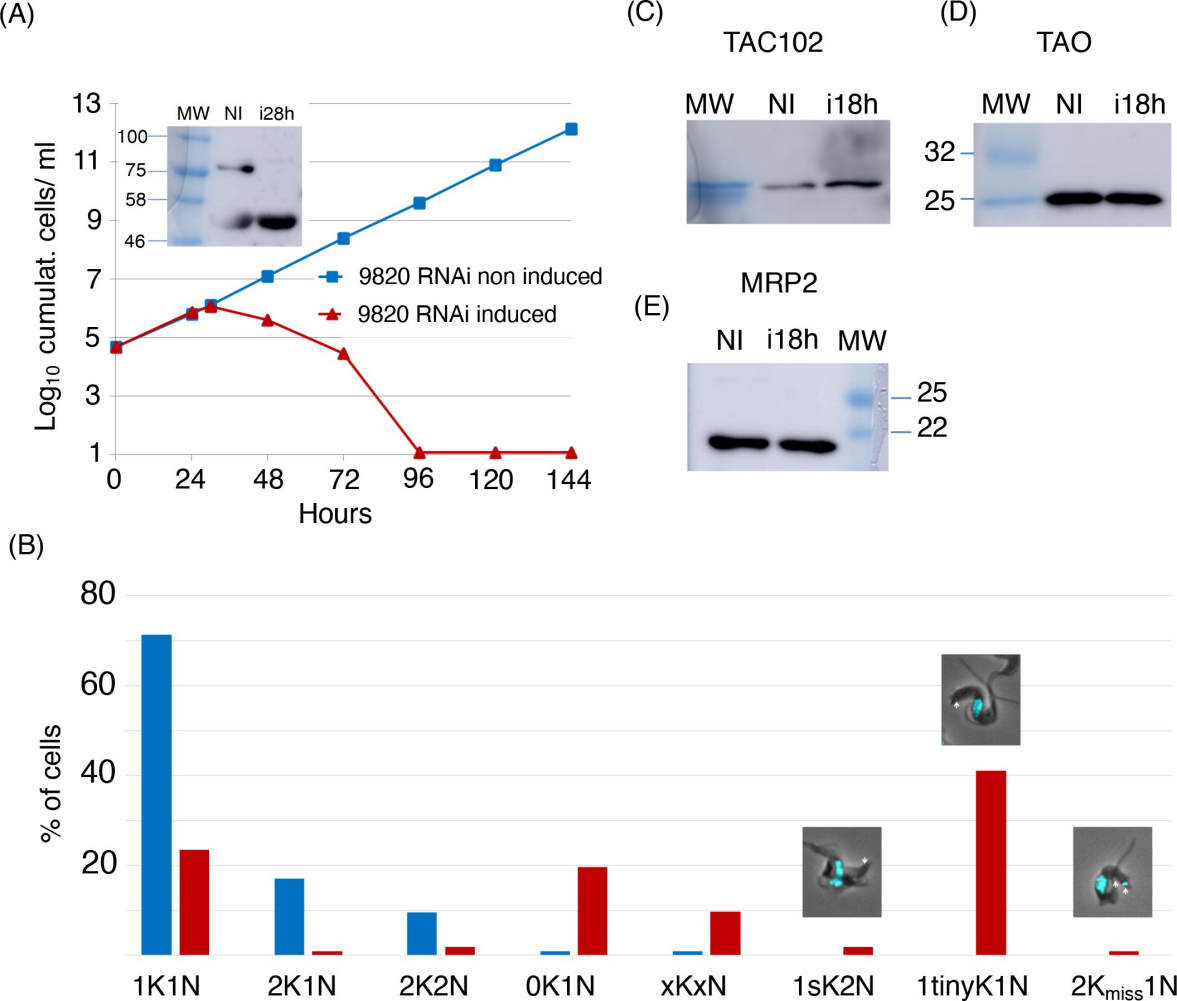
Characterisation of MIP RNAi cell line (BSF) (gene: Tb927.10.9820). (*A*) Growth curves of MIP RNAi cell line with induction (red) and without induction (blue) with tetracycline (n = 1). MIP was C-terminally Myc-tagged in the RNAi cell line. The fusion protein was expected at 80 kDa. The RNAi efficiency was assessed by western blot using an anti-Myc antibody (rabbit). The upper band corresponded to MIP-Myc. The lower band was an unspecific band detected by the antibody. (*B*) Cell cycle status of MIP RNAi cells (BSF) with (red) and without (blue) induction of RNAi for 24 h. Around 100 cells were observed per condition (non-induced and induced, n = 1). Exemplary composite pictures are shown on top of the columns for “1K2N small K”, “1K1N tiny K” and “2K1N misseg” categories. The kDNAs are indicated with white arrows. (*C*) Western blot of MIP RNAi cell line probed for TAC102. A 3.5% gel was used. (*D*) Western blot of MIP RNAi cell line probed for TAO. A 12% gel was used. (*E*) Western blot of MIP RNAi cell line probed for MRP2. A 12% gel was used. kDa: kilodalton; MW: molecular weight; NI: RNAi non-induced; i28h: RNAi induced during 28 h. n = 1 for all experiments.

TAC102 BioID identified MIP as a potential interactor of Myc-BirA*-TAC102. MIP is the mitochondrial intermediate peptidase, a protein which is part of the process of import of proteins into the mitochondrial matrix. It is encoded by the gene Tb927.10.9820. The protein was previously described to be essential in PCF cells [37]. RNAi was performed in BSF parasites and led to a strong growth defect, starting from two days of induction, and fastly leading to death of the cells (Fig 4A). After 24 hours of RNAi induction, we could see an increase of cells with no kDNA, small or tiny kDNA, multiples kinetoplasts and nuclei, or missegregated kinetoplasts (Fig 4B). In order to know if MIP was involved in processing TAC102, we investigated if the precursors of three mitochondrial matrix proteins (TAC102, TAO and MRP2) were detectable in MIP RNAi samples (Fig 4C, 4D and 4E). However no precursor accumulation was observed.

### TAC102 immunoprecipitation

**Fig 5:**
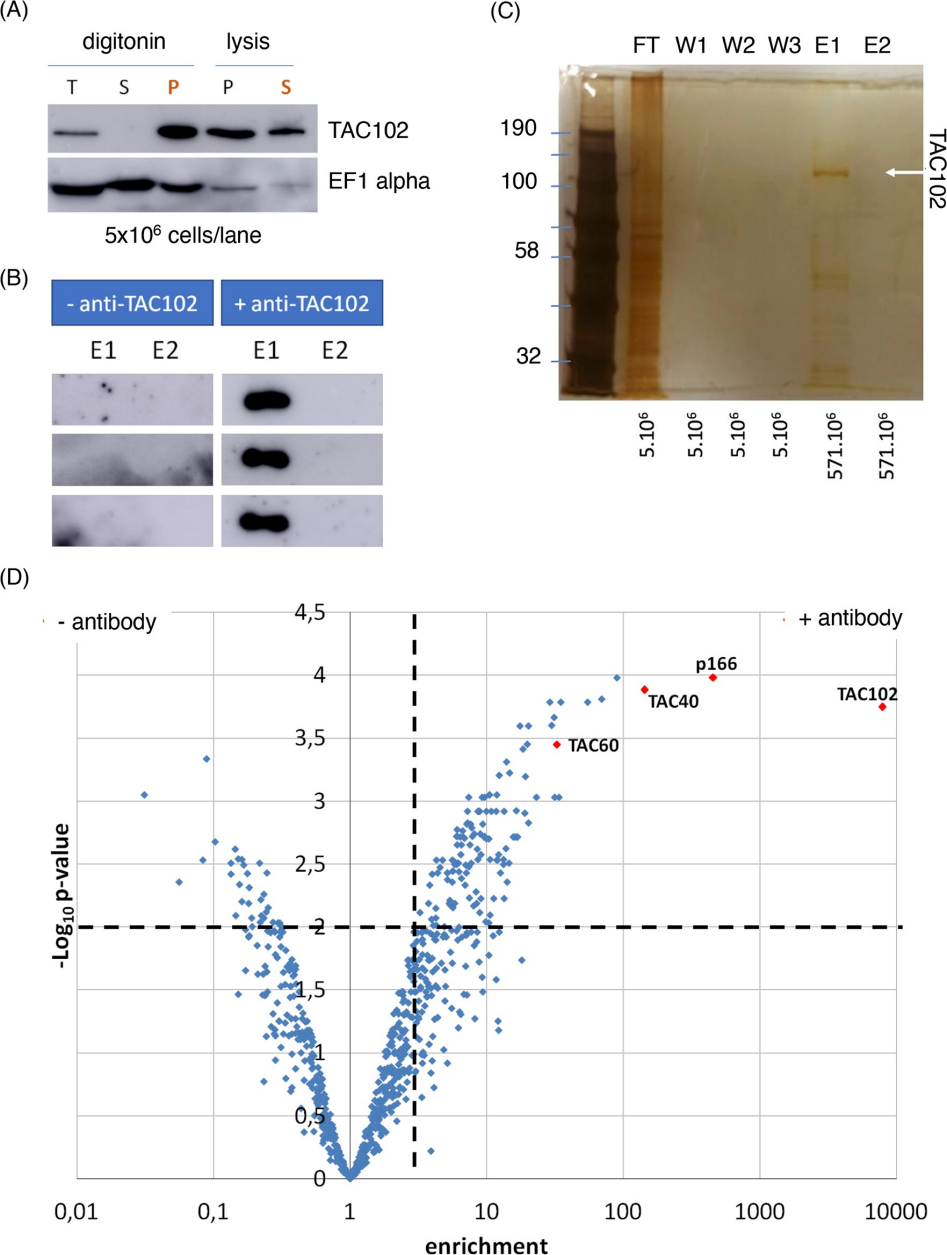
TAC102 enrichment and immunoprecipitation. The experiments were done in three independent replicates and all showed similar results. (*A*) Western blot analysis of the digitonin fractionation and subsequent lysis of the pellet fraction with 1% (v/v) Nonidet P-40 of whole cell protein from procyclic cells. T: total; S: supernatant; P: pellet. In orange: the pellet from the digitonin fractionation was used for the subsequent Nonidet P-40 lysis. The elongation factor EF1α served as a control. (*B*) Western blot analysis of the elutions (E1 and E2) of TAC102 immunoprecipitation replicates. (*C*) Silver stained SDS PAGE of TAC102 immunoprecipitation fractions. On the left are the molecular weights in kDa. Under each lane are written the equivalence of cells loaded. TAC102, indicated by a white arrow, was detected in E1. FT: flow-through; W1-W2-W3: washes; E1-E2: elutions. (*D*) Volcano plot representing the significance versus enrichment of the results from mass spectrometry analysis of TAC102 immunoprecipitation. A total of 775 proteins were identified from which 100 proteins were significantly enriched in the TAC102 immunoprecipitation (p ≤ 0.01). The two dotted lines represent the cut-off used (p ≤ 0.01 and enrichment > 3). The protein the most enriched in the condition was TAC102. Three other TAC components were also highly enriched (p166, TAC40 and TAC60).

In order to compare the BioID results with a more conventional approach, we used the monoclonal anti-TAC102 antibody coupled to magnetic beads for immunoprecipitation experiments. PCF cells were lysed with digitonin and fractionated into an organellar and cytoplasmic fraction by centrifugation. As shown previously, TAC102 was found in the organellar fraction [24]. After lysis of this fraction with 1% Nonidet P-40, about 50% of TAC102 was detected in the soluble fraction, which subsequently was used for immunoprecipitation (Fig 5A). The two elution fractions (E1 and E2) were resolved by SDS-PAGE and analysed by western-blot using the monoclonal anti-TAC102 antibody. TAC102 was enriched in the first elution step (E1) (Fig 5B) while the second elution fraction (E2) did not show a detectable amount of TAC102. In the fractions resolved on SDS-PAGE and stained with silver we found the majority of the proteins in the flowthrough (Fig 5C). The silver staining of the gel identified a prominent band in the elution (E1 fraction) between 100 kDa and 135 kDa, which likely corresponds to TAC102 itself (Fig 5C).

### TAC102 immunoprecipitation identifies TAC components

Immunoprecipitation with and without anti-TAC120 antibody coupled to the beads were done in triplicate. We used label free quantification methods to identify and quantify the peptides [38][39,40]. Enrichment was calculated, statistical significance was tested by an empirical Bayes test and the p-value was corrected by the Benjamin and Hochberg false discovery rate method, with a false discovery rate of 0.01. Of the 775 proteins that we detected, 100 proteins were enriched at least three-fold in the TAC102 immunoprecipitation (significance p ≤ 0.01) (Fig 5D, and S2 Table). Of these 100 proteins, 49 were predicted to have a mitochondrial localisation. TAC102 was the most enriched protein, followed by p166, another TAC component. Additionally, we detected two TAC components of the outer mitochondrial membrane, namely TAC40 and TAC60, among the top ten most enriched proteins. Nine of the 49 proteins with mitochondrial localisation were annotated as hypothetical proteins and four of these were in the top ten most enriched proteins. Aside from the hypothetical proteins and TAC components, we identified 30 translation factors, ten RNA binding proteins, seven DNA binding proteins, six ribosome biogenesis factors, two cristae formation proteins, two flagellum attachment zone proteins, two protein folding factors and two rRNA processing factors.

**Fig 6:**
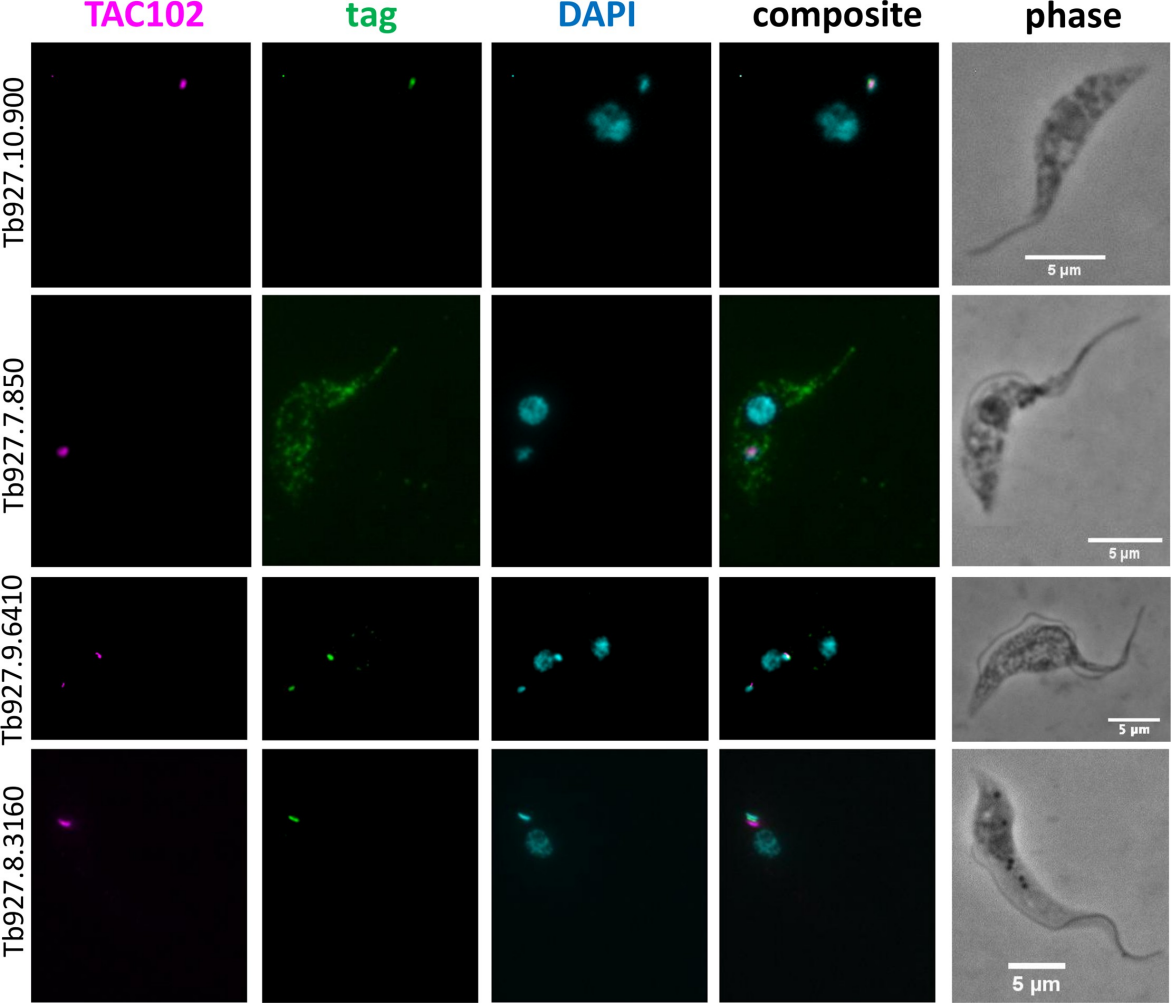
Localisations of the hypothetical proteins enriched significantly in both TAC102 BioID and TAC102 immunoprecipitation. Immunofluorescence microscopy was done in PCF. TAC102 was recognised by a monoclonal antibody. The candidate was HA tagged (Tb927.10.900, Tb927.7.850 and Tb927.8.3160) or MYC tagged (Tb927.9.6410) and was recognised by an anti-HA antibody or an anti-MYC antibody. DAPI stained the DNA content (nuclei and kinetoplasts). Around 100 cells were observed before choosing the example pictures.

When comparing the BioID and TAC102 immunoprecipitation data, we identified nine proteins to be significantly enriched in both approaches (Table 1). Aside from the bait TAC102, these were the mitochondrial protein import receptor ATOM69, two ribosomal proteins and five proteins with unknown function. In order to verify the localisation of the five proteins, we aimed to tag the corresponding genes in PCF trypanosomes *in situ* at the 3’ end, with a triple hemagglutinin or a myc tag, using a PCR based approach [41]. Three tagged proteins localised to the kDNA/TAC region (Tb927.10.900, Tb927.8.3160, Tb927.9.6410), one seemed to primarily localise in the cytoplasm (Tb927.7.850) and for one of the candidates we were unable to produce a C-terminally tagged cell line (Tb927.7.5330) (Fig 6).

**Table 1:** Nine proteins were enriched significantly in both TAC102 BioID and TAC102 immunoprecipitation (enrichment > 3; p ≤ 0.01).

Gene iD	Description	Functions	Enrichment	kDa	pI	Localisation	RNAi phen. pred.
Tb927.7.2390	TAC102	kDNA segregation	3949	103	9.42	TAC	+
Tb927.10.900	hyp.		98	19	10.95	cyto. (Nter)	+
Tb927.7.5330	hyp.		38	83	7.74	cyto. (Cter)	-
Tb927.7.850	hyp.		30	37	8.03		-
Tb927.9.6410	hyp.		28	75	7.51	TAC	-
Tb927.8.3160	hyp.		12	19	10.37		+
Tb927.4.4600	MRPL43	translation	10	31	10.1	mito.	-
Tb927.11.11460	ATOM69	mito. import	9	69	5.28	MOM	-
Tb927.8.5280	MRPS34	translation	7	29	5.41	mito.	+

Abbreviations:

cyto.: cytoplasm; hyp.: hypothetical protein; mito.: mitochondrial; MOM: mitochondrial outer membrane; pI: isoelectric point; MW: molecular weight; RNAi phen. pred.: RNAi phenotype prediction.

### Yeast two-hybrid screen shows interaction between TAC102 and p166

The yeast two-hybrid screen (Hybrigenics) was done essentially as described previously [35]. The entire open reading frame of TAC102 was fused to the C-terminus of LexA (N-LexA-TAC102) and expressed in yeast cells. A total of 138 million interactions were screened and 15 clones were isolated and characterised (S3 Table). Three high confidence hits were identified expressing a N-terminal part of the TAC component p166 ([22,28]). This large acidic TAC protein contains a single transmembrane domain in the C-terminus and was previously shown by superresolution microscopy to be in close proximity to TAC102 [22,28]. Two of the three high confidence p166 clones expressed a short region from amino acid 37 to 210 while the third clone expressed a larger fragment ranging from amino acid 71 to 716 of the 1502 amino acid long protein (S1 Fig). The only other hits with good confidence were two identical clones of the C-terminal region of the putative nuclear pore component NUP109, that was previously found in a proteomics analysis characterising nuclear proteins [42].

## Discussion

In this study, we employed three different approaches with the aim to identify interacting proteins of TAC102, which is a key component of the mitochondrial genome segregation machinery. We identified at least three potential novel TAC components and could establish the direct interaction of TAC102 and p166.

**Fig 7:**
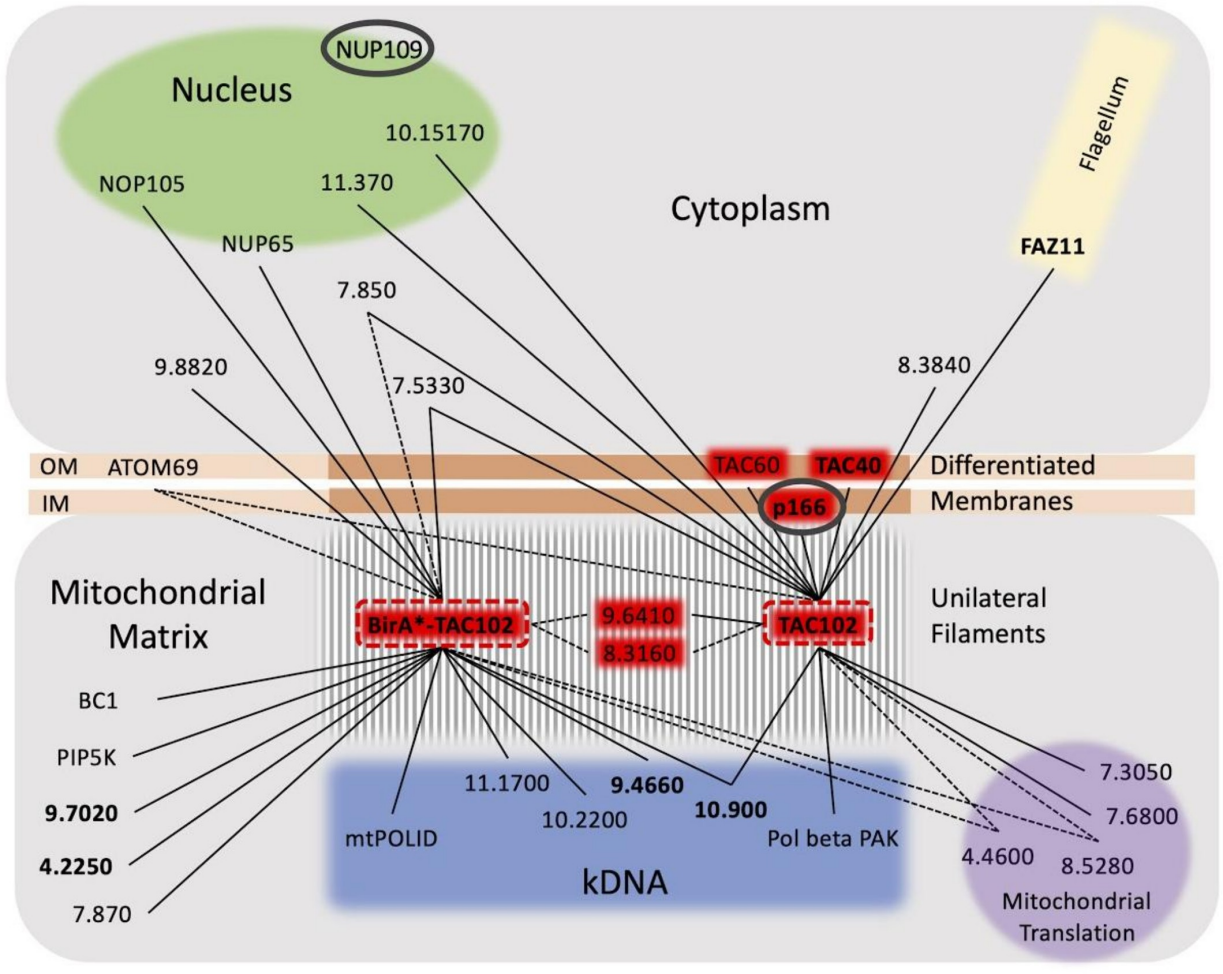
Depiction of the TAC102 interactions identified from BioID, immunoprecipitation and a yeast two hybrid screen. Gene identities of the protein of interest are shown here as chromosome and location coordinates only. The common interactors (BioID/CoIP) are connected with a dotted line (——). The 15 most enriched proteins of the TAC102 IP or BirA*-TAC102 (BioID) are connected with a solid line (___). The five most enriched proteins are in **bold**. The two proteins identified by the yeast two hybrid screen are circled in grey (⬭). BC1: cytochrome b-c1 subunit; IM: inner mitochondrial membrane; NOP: nucleolar protein; NUP: nucleoporin; OM: outer mitochondrial membrane; PIP5K: phosphatidylinositol-4-phosphate 5-kinase related; POLID: DNA polymerase I D; Pol beta PAK: Polymerase beta with PAK domain.

One of the approaches applied proximity-dependent biotin identification (BioID), which was previously used by Morriswood and colleagues for the identification of proteins localised in the region of the hook complex in *T*. *brucei* [33]. While there are a few studies using BioID in combination with mitochondrially targeted proteins, like the protein interaction study on Clp in human 293T cells [43] or the mitochondrial PolG interactome [44], in this study we show for the first time the potential of BioID with a mitochondrial protein of a protist from the group of the excavates. Although we did not formally test if the Myc-BirA*-TAC102 fusion protein was capable of rescuing a TAC102 knockout, the localisation by immunofluorescence microscopy (see Fig 1A), the biochemical behavior during detergent solubilisation (Fig 1B) and the lack of a dominant negative phenotype argue that the protein is likely assembled in the TAC. The detection of multiple signals for TAC102/Myc-BirA*-TAC102 on western blot after the biochemical fractionation is potentially due to the instability of TAC102 when released from the TAC structure. Most of the biotinylated proteins detected by fluorescence microscopy (Fig 2B) localised close to the kDNA, however, there was also some signal visible outside this area. At this point we can only speculate if this was a non-specific staining or true, potentially transient interactors of Myc-BirA*-TAC102. Furthermore, we can also not exclude that a weak overexpression of Myc-BirA*-TAC102 led to a partial mislocalisation and corresponding labelling of proteins not found in the TAC. Nevertheless, consistent with the localisation of the fusion construct Myc-BirA*-TAC102 in the mitochondrial organelle, most of the identified interactors (> 60%) were known mitochondrial proteins and half of those were known to localise at the kDNA or the TAC itself (S1 Table). While the identification of kDNA associated proteins was not surprising since they are in close proximity to the TAC, we also identified two components of the mitochondrial protein import machinery. One was ATOM69, a receptor of the major import pore, the archaic translocase of the mitochondrial outer membrane (ATOM) in trypanosomes [45]. In most cases, protein import into the mitochondrion requires an N-terminal targeting signal. The Myc-BirA*-TAC102 fusion protein however uses its natural import signal that was previously shown to reside in the C-terminal region of TAC102 [24]. Thus, unfolding and import from the C-terminus of the fusion construct would allow for biotinylation of the receptor during the import process. The second protein that is part of the import process and seems to interact with Myc-BirA*-TAC102 was the mitochondrial intermediate peptidase MIP, which was previously described to be essential in PCF cells [37]. We asked if depletion of the peptidase would lead to TAC102 precursor accumulation. RNAi targeting the mitochondrially localised MIP in BSF cells led to a very strong growth defect, however no precursor accumulation of TAC102 was observed (Fig 4). Thus, either there is no processing and the interaction of TAC102 and MIP serves a different purpose, or the processed peptide is very short and not readily detectable, or the MIP was a false positive interaction. Compared to the BioID approach, immunoprecipitation using a monoclonal TAC102 antibody provided the advantage of targeting the native protein rather than relying on an artificial fusion protein. However, the immunoprecipitation with TAC102 as bait did not lead to a greater enrichment of mitochondrial proteins, when compared to the BioID approach and in both cases TAC102 was the most highly enriched protein. Interestingly, the TAC102 immunoprecipitation identified the TAC component p166 as the second most enriched protein (after TAC102), while BioID did not detect any currently characterised TAC component except TAC102 itself. The interaction of TAC102 and p166 was confirmed by the yeast two-hybrid screen which demonstrated that the TAC102 interaction domains are in the N-terminal region of p166 (S1 Fig, S3 Table). Thus, the C-terminus of p166 with its single predicted transmembrane domain is likely to reside at the inner mitochondrial membrane, while the N-terminus connects to the kDNA proximal TAC102. So, if p166 is a direct interactor of TAC102, why did the immunoprecipitation, but not the BioID approach, identify this interaction? One explanation could be the orientation of the BirA*-TAC102 fusion protein in the TAC. If TAC102 is interacting with p166 via its C-terminus, then the BirA* moiety of the fusion protein might be facing the kDNA and be too far away from p166 for proximity labelling. This would also explain why the BioID approach identified seven proteins close or in the kDNA network. Alternatively, the BirA*-TAC102 fusion protein, despite its apparent correct localisation, might not be incorporated properly into the TAC and thus not be in proximity to p166. Another explanation could also be that the elution from the streptavidin-conjugated beads, by boiling in the Laemmli buffer, was not efficient. Therefore, some proteins were remaining on the beads and could not be identified by mass spectrometry. Aside from p166, the TAC102 immunoprecipitation also enriched two TAC components of the outer mitochondrial membrane (TAC40 and TAC60, Fig 7), which are in close proximity to p166 in the TAC [28]. Both approaches (BioID and IP) did enrich a known component of the kDNA replication machinery (Fig 7). TAC102-BioID identified the mitochondrial PolID, which dynamically localises at the antipodal sites during minicircle replication [46]. TAC102 immunoprecipitation identified Pol beta PAK, a polymerase localised throughout the kDNA disc, which is believed to be involved in minicircle gap closure prior to segregation of the network [47]. Since replication of the minicircles occurs in the kinetoflagellar zone, which is also the home of the TAC, it is not surprising to find at least some of the replication components interacting with the segregation machinery (Fig 7). In conclusion, we have for the first time used BioID to identify interactors of a mitochondrial protein in trypanosomes and compared the approach to immunoprecipitation and yeast two-hybrid. We were able to identify three proteins that, by their localisation, could potentially represent novel TAC components and established the direct interaction of TAC102 with the N-terminal region of p166.

## Material and methods

### Antibodies and reagents

The rabbit polyclonal anti-Myc antibody and the biotin was purchased from Sigma-Aldrich. The mouse anti-EF1α antibody was purchased from Santa Cruz Biotechnology. The mouse monoclonal anti-TAC102 antibody and the rabbit anti-ATOM antibody have been described previously [24,48]. Streptavidin-conjugated magnetic beads were purchased from NEB. HRP-conjugated streptavidin and AlexaFluor488-conjugated streptavidin were purchased from Thermo Fisher Scientific.

### Trypanosomes, culture and generation

We cultured procyclic form (PCF) Lister 427 29–13 and bloodstream form (BSF) Lister 427 SM, *Trypanosoma brucei brucei* as described previously [49]. In brief, PCF/BSF cells were grown at 27°C/37°C without and with 5% (v/v) CO_2_, in SDM79/HMI9 medium supplemented with 10% (v/v) of fetal calf serum (Sigma-Aldrich).

To generate the PCF Myc-BirA*-TAC102 cell line, parasites were transfected with the *Not*I linearised pLew100_Myc_BirA* plasmid containing the coding sequence of TAC102. Blasticidin at the concentration of 10 μg/mL was added in the medium for selection. Myc-BirA*-TAC102 expression was induced by tetracycline (1 μg/mL).

To generate the BSF MIP RNAi cell line, parasites were transfected with the *Not*I linearised pFC-4 plasmid containing a sense-antisense construct, under control of a tetracycline operator. MIP RNAi was then induced by tetracycline (1 μg/mL).

To generate the C-terminally tagged cell lines, parasites were transfected with a clean PCR product made with a pMOTag plasmid as template [41].

BSF trypanosomes (4x10^7^ cells) in exponential phase were transfected with 10 μg of DNA in transfection buffer (90 mM Na-phosphate (387 mM Na_2_HPO_4_, 113 mM NaH_2_PO_4_) pH 7.3, 5 mM KCl, 0.15 mM CaCl_2_, 50 mM HEPES pH 7.3). Transfections were carried out with an electric shock (one pulse, programme Z-001, [50]) using the AMAXA Nucleofector II (Lonza). Parasites were then diluted into three 24-well plates to get 10^5^ cells/mL, 10^4^ cells/mL and 10^3^ cells/mL. Around 20 hours later, the selection antibiotic was added. Clones emerged around five days after adding the selection antibiotic. One clone was used for downstream analyses.

PCF trypanosomes (1x10^7^ cells) in exponential phase were transfected with 1 μg of DNA essentially as described previously [51]. Transfections were carried out with an electric shock (one pulse at 1500 V, 25 μF, 186 Ω, 2.5 kV/resistance). The next day, the selection antibiotic was added and the pool of transfection was serial diluted in a 96-well plate, with a ½ dilution in between each lane. The clones emerged around ten to 15 days later. One clone was used for downstream analyses.

### Wide field fluorescence microscopy

5x10^5^ cells were allowed to settle on a slide for 40 min. The cells were fixed for four minutes with 4% (m/v) paraformaldehyde solution (PFA 4%) in PBS. Cells were then permeabilised with 0.2% (v/v) Triton X-100 for five minutes. After each treatment, the slide was washed with PBS. The slide was then blocked in PBS-BSA 4% (m/v) for 30 min in a humid atmosphere. The primary antibodies and secondary antibodies were added to the slide and incubated for one hour each at room temperature. The primary and secondary antibodies were diluted in PBS-BSA 4% (anti-Myc rabbit 1/1000, anti-TAC102 mouse 1/5000). Post staining cells were mounted in ProLong Gold antifade reagent (Life technologies) containing 4′,6-diamidino-2-phenylindole (DAPI).

The slides were observed with a 100x oil immersion phase contrast objective on the Leica DM 5500 fluorescence microscope. LAS X software (Leica) was used for acquisition of pictures. Fiji (ImageJ) was used to process and analyse the pictures.

### SDS-PAGE

Denaturing polyacrylamide gels (mix acrylamide/bis-acrylamide, Tris, SDS), composed of a resolving (Tris pH 6.8) and a stacking gel (Tris pH 8.8), were made and polymerised with ammonium persulfate (APS) and tetramethylethylenediamine (TEMED). Gels were run in SDS running buffer (25 mM Tris, 192 mM glycine, 0.1% (v/v) SDS) at 80 V for around two hours (for an 8% (m/v) resolving gel).

For mass spectrometry analyses, samples were run on a 10% precast SDS gel (BioRad) for two minutes at 200 V.

### Coomassie staining

Gels were incubated for at least one hour in Coomassie (40% (v/v) methanol, 10% (v/v) acetic acid, 0.25% (m/v) Coomassie blue R250) and then destained for at least two hours in destaining solution (40% (v/v) methanol, 10% (v/v) acetic acid).

For mass spectrometry analyses, gels were stained with Brilliant Blue R-250 Coomassie (BioRad) for at least one hour and then destained with Coomassie Brilliant Blue R-250 Destaining Solution (BioRad) for at least two hours.

### Silver staining

Gels were incubated in 5% (v/v) methanol, 7% (v/v) acetic acid for one hour, then in 5% (v/v) glutaraldehyde for 15 min. They were rinsed at least four times for 15 min with water. Gels were then incubated in a solution of 32 μM dithiothreitol (DTT) for 15 min, then in a solution of 0.1% (m/v) AgNO_3_ for 15 min. After quick rinses with water, gels were developed using a solution of 283 mM Na_2_CO_3_, 0.0185% (v/v) formaldehyde. Reaction was stopped using 48% citric acid.

### BioID

The primers used for the amplification of TAC102 open reading frame were 2390 BamHI fwd: 5’- CGGGATCCATGTATCGGCCTCGTGGCGG -3’ 2390 SalI rev: 5’- CGGGTCGACTTACTTTATAAGCTGCCGAA -3’

The sequence coding for TAC102 was cloned in between the restriction sites of the enzymes *Xho*I and *BamH*I into the pLew100_Myc_BirA* vector [33]. The protocol used was an adaptation of the one described in [33]. 2.5 mL of biotin stock solution (1 mM in sterile MilliQ water) was added to 47.5 mL of PCF cells and incubated for 24 h, in order to give biotin time to enter the cells. The expression of Myc-BirA*-TAC102 was subsequently induced for six hours with tetracycline. We used 5x10^8^ cells per experiment. They were collected by centrifugation (1800 g, 5 min, 4°C), washed three times with phosphate-buffered saline (PBS: 137 mM NaCl, 2.7 mM KCl, 10 mM Na_2_HPO_4_, 2 mM KH_2_PO_4_) and finally resuspended in PEME buffer (2 mM EGTA, 1 mM MgSO_4_, 0.1 mM EDTA, 0.1 M PIPES, pH 6.9) containing 0.5% (v/v) Nonidet P-40 and protease inhibitors (cOmplete ULTRA Tablets, Mini, EDTA-free, Roche). The tube was left for 15 min at room temperature with gentle mixing: this was the E1 fraction (see Fig 3A). The tube was then centrifuged (3400 g, 2 min, RT) and the supernatant was put in a new tube: this was the S1 fraction. The pellet was then resuspended in a lysis buffer (0.4% (m/v) SDS, 500 mM NaCl, 5 mM EDTA, 1 mM DTT, 50 mM Tris-HCl pH 7.4). The tube was left for 30 min at room temperature with a gentle mixing: this was the P1 fraction. After centrifugation (16 000 g, 10 min, RT), the supernatant was put in a new tube: this was the S2 fraction. 250 μL of streptavidin conjugated magnetic beads (NEB) were added to S1 and S2. The tubes were then incubated for four hours at 4°C before separation of the beads from the liquid with a magnet: these were fractions F1 and F2. The beads were then washed twice with PBS, centrifuged (6000 g, 2 min, RT) and finally resuspended in Laemmli buffer (5X stock solution: 2% (m/v) SDS, 60 mM Tris-HCl pH 6.8, 24% (v/v) glycerol, 5% (v/v) β-mercaptoethanol, bromophenol blue). The beads and fractions from each sample were boiled in Laemmli buffer for 5 min before being separated by SDS-PAGE, transferred to polyvinylidene difluoride (PVDF) membrane and blotted with HRP-conjugated streptavidin.

For mass spectrometry analysis, beads were run on a 10% precast SDS gel (BioRad) for two minutes at 200 V. The gel was stained with Brilliant Blue R-250 Coomassie (BioRad) for at least one hour and then destained with Coomassie Brilliant Blue R-250 Destaining Solution (BioRad) for at least two hours. The bands were then cut out and sent to mass spectrometry analysis for trypsin digest and nanoLC-MS/MS at the Proteomics Mass Spectrometry Core Facility (PMSCF) of the University of Bern. Protein identification was done using the MaxQuant software package and the genome sequences of the *T*. *brucei* 427 and 927 strains essentially as described previously [52].

### Immunoprecipitation

For the immunoprecipitation of TAC102, anti-TAC102 antibody was crosslinked to protein A / protein G beads from the Pierce Crosslink Magnetic IP kit (Thermo Scientific). A mitochondrial enriched fraction was prepared by digitonin fractionation with 100 mL of procyclic form parasites (cell line: 29.13) grown in the exponential phase. The mitochondrial enriched fraction was then resuspended in ice-cold IP lysis/wash buffer (25 mM Tris, 150 mM NaCl, 1 mM EDTA, 1% (v/v) Nonidet P-40, 5% (v/v) glycerol) containing protease inhibitors. The sample was incubated on ice for five minutes, with vortexing every minute. After centrifugation (13 000 g, 10 min, 4°C), the supernatant was kept and put on beads containing anti-TAC102 monoclonal antibodies or empty beads (for negative controls), for 1:35 h at room temperature with rotation. The mixture lysate/beads was vortexed every 15 min during this incubation. The beads were collected on a magnetic stand for one minute and the flow-through (FT) was kept on ice in a new tube. The beads were washed twice with ice-cold IP lysis/wash buffer containing protease inhibitors and once with ice-cold water containing protease inhibitors. The proteins bound to the beads were then eluted twice using 300 μL of 0.1 M glycine pH 2.4 containing protease inhibitors with a five minutes incubation on a rotating platform. The elutions were saved on ice in new tubes and the pH was neutralised using 30 μL of neutralisation buffer from the kit. In order to get a reasonable amount of proteins to load on a gel for further mass spectrometry analysis, the elutions from immunoprecipitations were acetone precipitated. To do so, four times the sample volume of cold acetone (-20°C) was added into the elutions. The tube was vortexed and incubated for at least one hour at -20°C. After centrifugation (15 000 g, 10 min, 4°C), the supernatant was removed and the rest of the acetone was allowed to evaporate for 30 min at room temperature. The protein pellet was then resuspended in 20 μL of 1x Laemmli buffer, boiled for five minutes and run on a 10% precast SDS gel (BioRad) for two minutes at 200 V. The gel was stained with Brilliant Blue R-250 Coomassie (BioRad) for at least one hour and then destained with Coomassie Brilliant Blue R-250 Destaining Solution (BioRad) for at least two hours. The bands were then cut out and sent to mass spectrometry analysis for trypsin digest and nanoLC-MS/MS at the Proteomics Mass Spectrometry Core Facility (PMSCF) of the University of Bern. Identification was done as described above.

### Yeast two-hybrid (Y2H)

Bait cloning and Y2H screening were performed by Hybrigenics Services SAS, France (http://hybrigenics.com/services). The coding sequence for full-length TAC102 (Tb927.7.2390) was cloned into a plasmid pB27 as a C-terminal fusion to LexA (N-Lex-TAC102). The construct was used as a bait to screen at saturation a highly complex Treu927 genomic fragment library of *T*. *brucei* constructed into pP6. pB27 and pP6 derive from the original pBTM116 and pGADGH plasmids, respectively. 138 million clones were screened using a mating approach with Y187 (matα) and L40٨Gal4 (mata) yeast strains as previously described [53]. 15 His+ colonies were selected on a medium lacking tryptophan, leucine and histidine. The prey fragments of the positive clones were amplified by PCR and sequenced. The resulting sequences were used to identify the corresponding interacting proteins in *Trypanosoma brucei* Treu927 genome sequence using a fully automated procedure in the GenBank database (NCBI). A confidence score (PBS, for Predicted biological score) was attributed to each interaction as previously described [54]. This global score represents the probability of an interaction being nonspecific. The PBS scores have been used to select out of the 15 clones those with high confidence interaction (Score B).

Biochemical methods including digitonin fractionation, Coomassie and silver stained gels were done as described previously [49]. For mass spectrometry analysis the samples were briefly separated on precast 10% polyacrylamide gels under denaturing conditions. Pieces of the gel were then treated essentially as described previously [52].

## Supporting information

S1 Fig TAC102 yeast two-hybrid screen high confidence interactions. Depicted are the three high confidence interaction clones (Y2H-Clones, dark grey) that express a N-terminal region of p166. p166 (light grey) is shown as reference including the C-terminal predicted transmembrane (TM) domain. The region sufficient for TAC102 interaction is shown in yellow.
(DOCX)

S1 Table Enriched proteins from Myc-BirA*-TAC102 BioID (enrichment > 2; p ≤ 0.01). Abbreviations: ADKA: adenylate kinase; act.: activity; ALAT: alanine aminotransferase; BB: basal body; bind.: binding; c. b-c1 su.: cytochrome b-c1 subunit; cont.: containing; CPC: chromosomal passenger complex; DUF: domain of unknown function; ER: endoplasmic reticulum; flagel.: flagellar; GRBC: guide-RNA binding complex; hyp.: hypothetical protein; IF: initiation factor; iso. p.: isoelectric point; KAP: kDNA-associated protein MIP: mitochondrial intermediate peptidase; mito.: mitochondrial; mol.: molecule; MRP: mitochondrial ribosomal protein; mt. SSU r.: mitochondrial small subunit ribosomal; NDUF: NADH-ubiquinone oxidoreductase subunit; NOP: nucleolar protein; nucl.: nucleus; nucleol.: nucleolus; NUP: nucleoporine; PIP5K: phosphatidylinositol-4-phosphate 5-kinase related; POLID: mitochondrial DNA polymerase I D; pr. RanGDP b.: predicted RanGDP binding; prot.: protein; put: putative; SIM: SUMO-interacting motif; SRP: signal recognition particle; struct.: structural; ter.: terminal.
(DOCX)

S2 Table Proteins enriched in TAC102 immunoprecipitation (enrichment > 2; p ≤ 0.01). Abbreviations: AKAP: A-kinase anchor protein; bind.: binding; cAMP: cyclic adenosine monophosphate; cyto.: cytoplasmic; dehyd.: dehydrogenase; DUF: domain of unknown function; eIF: eukaryotic initiation factor; ER: endoplasmic reticulum; FAZ: flagellum attachment zone; glycos.: glycosomal; iso. p.: isoelectric point; isom.: isomerase; methyltransf.: methyltransferase; MICOS: mitochondrial contact site and cristae organisation system; mito.: mitochondrial; MRP: mitochondrial ribosomal protein; mt. SSU: mitochondrial small subunit ribosomal; NOP: nucleolar protein; nucl.: nucleus; nucleol.: nucleolus; prot.: protein; put: putative; r.: ribosomal; RRS1: ribosome biogenesis regulator protein 1; snRNP: small nuclear ribonucleoprotein; struct.: structural; s.u.: subunit; ter.: terminal.
(DOCX)

S3 Table TAC102 yeast two-hybrid screen clones. (DOCX)

S1 Data Contains the numerical data used in this manuscript. (XLSX)
