# Supplementary material for: A comparison of three approaches for the discovery of novel tripartite attachment complex proteins in Trypanosoma brucei
Source: PLoS Negl Trop Dis. 2020 Sep 16;14(9):e0008568. doi: 10.1371/journal.pntd.0008568 (PMC7521757; doi:10.1371/journal.pntd.0008568)
Supplement: S1 Fig — Depicted are the three high confidence interaction clones (Y2H-Clones, dark grey) that express a N-terminal region of p166. p166 (light grey) is shown as reference including the C-terminal predicted transmembrane (TM) domain. The region sufficient for TAC102 interaction is shown in yellow. (DOCX) [file pntd.0008568.s001.docx]

**Supplementary information**


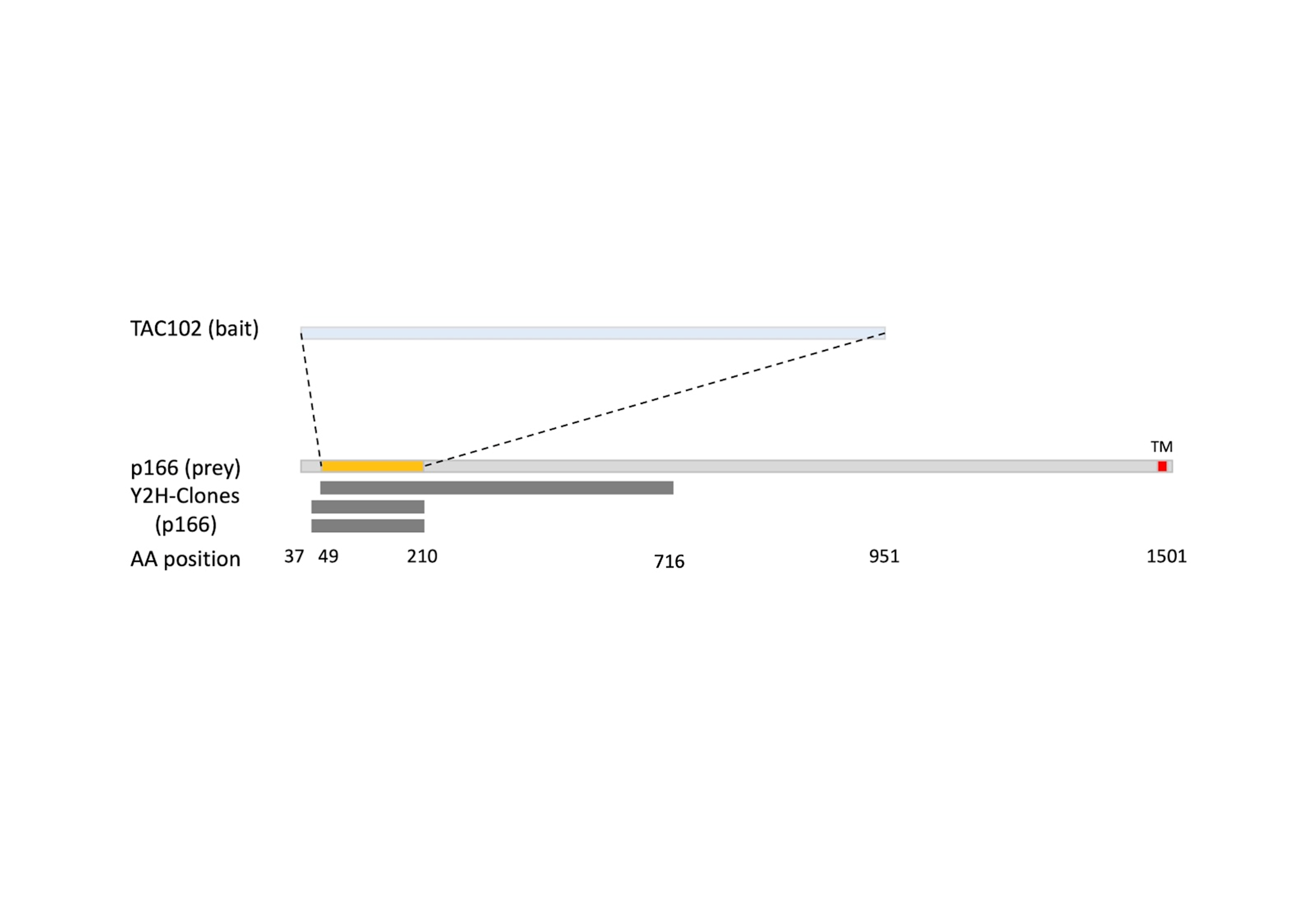


**Fig S1. TAC102 yeast two-hybrid screen high confidence interactions.** Depicted are the three high confidence interaction clones (Y2H-Clones, dark grey) that express a N-terminal region of p166. p166 (light grey) is shown as reference including the C-terminal predicted transmembrane (TM) domain. The region sufficient for TAC102 interaction is shown in yellow.
