# Supplementary material for: A comparison of three approaches for the discovery of novel tripartite attachment complex proteins in Trypanosoma brucei
Source: PLoS Negl Trop Dis. 2020 Sep 16;14(9):e0008568. doi: 10.1371/journal.pntd.0008568 (PMC7521757; doi:10.1371/journal.pntd.0008568)
Supplement: S1 Table — Abbreviations: ADKA: adenylate kinase; act.: activity; ALAT: alanine aminotransferase; BB: basal body; bind.: binding; c. b-c1 su.: cytochrome b-c1 subunit; cont.: containing; CPC: chromosomal passenger complex; DUF: domain of unknown function; ER: endoplasmic reticulum; flagel.: flagellar; GRBC: guide-RNA binding complex; hyp.: hypothetical protein; IF: initiation factor; iso. p.: isoelectric point; KAP: kDNA-associated protein MIP: mitochondrial intermediate peptidase; mito.: mitochondrial; mol.: molecule; MRP: mitochondrial ribosomal protein; mt. SSU r.: mitochondrial small subunit ribosomal; NDUF: NADH-ubiquinone oxidoreductase subunit; NOP: nucleolar protein; nucl.: nucleus; nucleol.: nucleolus; NUP: nucleoporine; PIP5K: phosphatidylinositol-4-phosphate 5-kinase related; POLID: mitochondrial DNA polymerase I D; pr. RanGDP b.: predicted RanGDP binding; prot.: protein; put: putative; SIM: SUMO-interacting motif; SRP: signal recognition particle; struct.: structural; ter.: terminal. (DOCX) [file pntd.0008568.s002.docx]

**Table S1. Enriched proteins from Myc-BirA*-TAC102 BioID (enrichment > 2 ; p ≤ 0.01).**


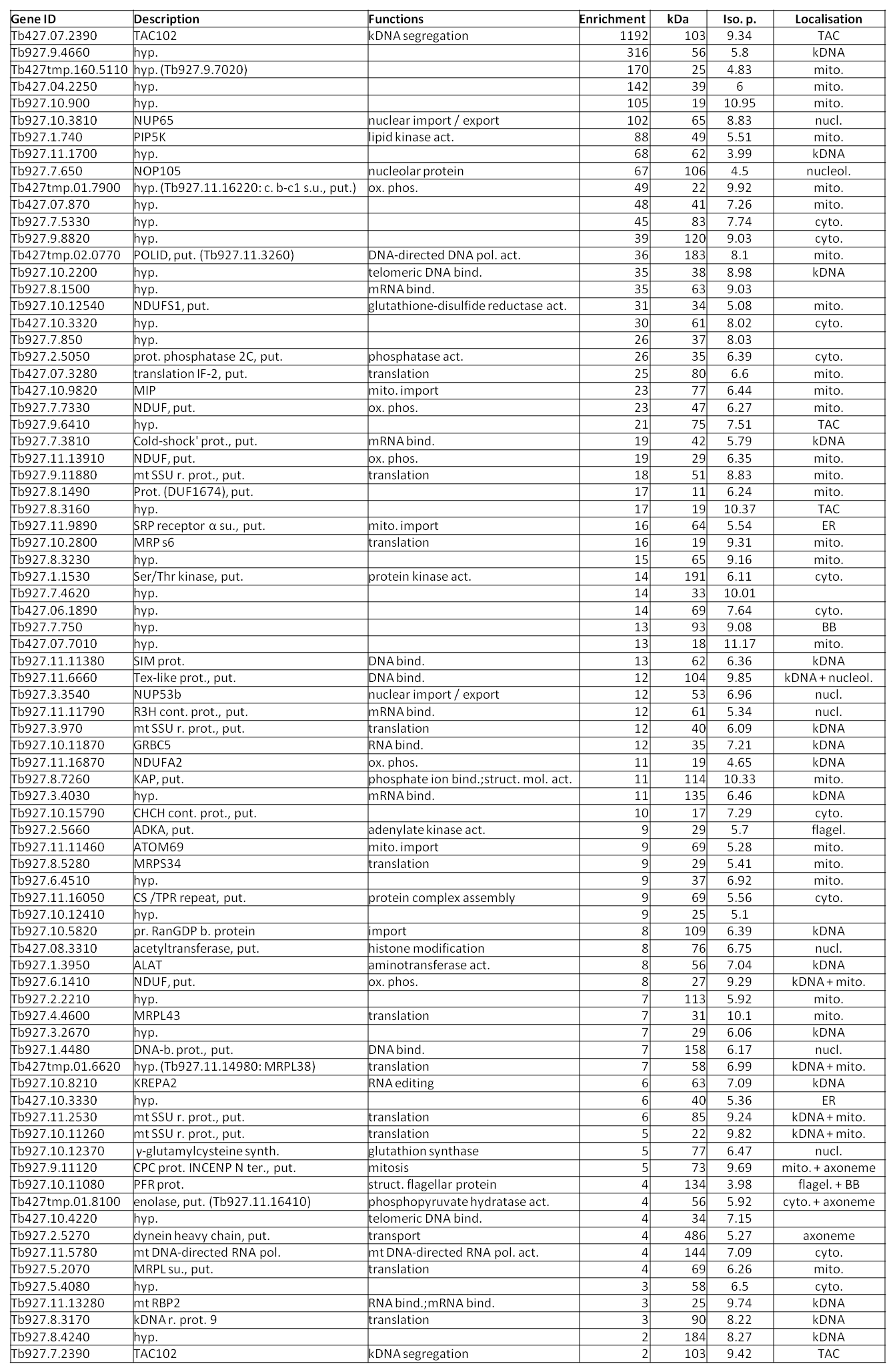


Abbreviations:

ADKA: adenylate kinase; act.: activity; ALAT: alanine aminotransferase; BB: basal body; bind.: binding; c. b-c1 su.: cytochrome b-c1 subunit; cont.: containing; CPC: chromosomal passenger complex; DUF: domain of unknown function; ER: endoplasmic reticulum; flagel.: flagellar; GRBC: guide-RNA binding complex; hyp.: hypothetical protein; IF: initiation factor; iso. p.: isoelectric point; KAP: kDNA-associated protein MIP: mitochondrial intermediate peptidase; mito.: mitochondrial; mol.: molecule; MRP: mitochondrial ribosomal protein; mt. SSU r.: mitochondrial small subunit ribosomal; NDUF: NADH-ubiquinone oxidoreductase subunit; NOP: nucleolar protein; nucl.: nucleus; nucleol.: nucleolus; NUP: nucleoporine; PIP5K: phosphatidylinositol-4-phosphate 5-kinase related; POLID: mitochondrial DNA polymerase I D; pr. RanGDP b.: predicted RanGDP binding; prot.: protein; put: putative; SIM: SUMO-interacting motif; SRP: signal recognition particle; struct.: structural; ter.: terminal
