# Supplementary material for: A comparison of three approaches for the discovery of novel tripartite attachment complex proteins in Trypanosoma brucei
Source: PLoS Negl Trop Dis. 2020 Sep 16;14(9):e0008568. doi: 10.1371/journal.pntd.0008568 (PMC7521757; doi:10.1371/journal.pntd.0008568)
Supplement: S2 Table — Abbreviations: AKAP: A-kinase anchor protein; bind.: binding; cAMP: cyclic adenosine monophosphate; cyto.: cytoplasmic; dehyd.: dehydrogenase; DUF: domain of unknown function; eIF: eukaryotic initiation factor; ER: endoplasmic reticulum; FAZ: flagellum attachment zone; glycos.: glycosomal; iso. p.: isoelectric point; isom.: isomerase; methyltransf.: methyltransferase; MICOS: mitochondrial contact site and cristae organisation system; mito.: mitochondrial; MRP: mitochondrial ribosomal protein; mt. SSU: mitochondrial small subunit ribosomal; NOP: nucleolar protein; nucl.: nucleus; nucleol.: nucleolus; prot.: protein; put: putative; r.: ribosomal; RRS1: ribosome biogenesis regulator protein 1; snRNP: small nuclear ribonucleoprotein; struct.: structural; s.u.: subunit; ter.: terminal. (DOCX) [file pntd.0008568.s003.docx]

**Table S2.** **Proteins enriched in TAC102 immunoprecipitation (enrichment > 2 ; p ≤ 0.01).**
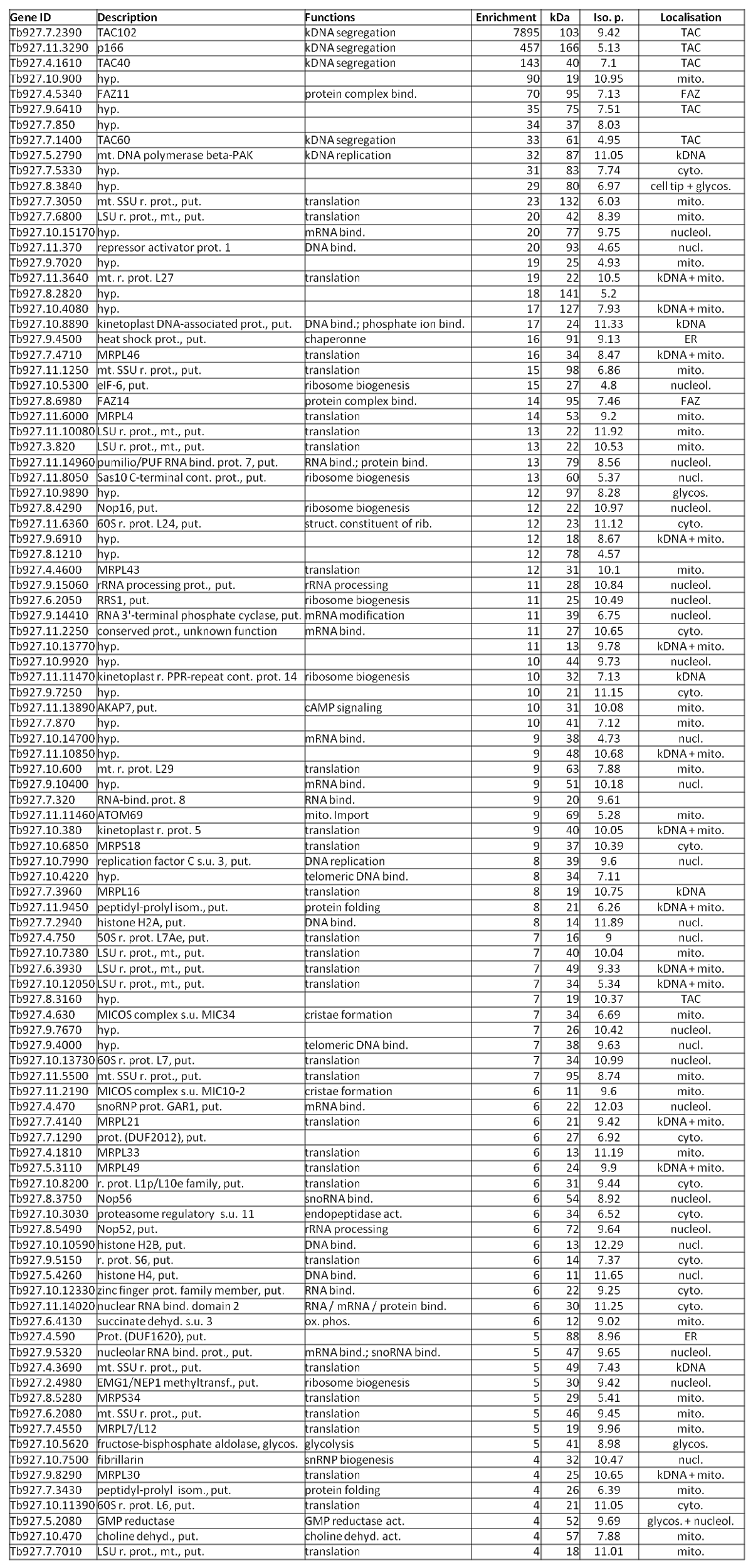


Abbreviations:

AKAP: A-kinase anchor protein; bind.: binding; cAMP: cyclic adenosine monophosphate; cyto.: cytoplasmic; dehyd.: dehydrogenase; DUF: domain of unknown function; eIF: eukaryotic initiation factor; ER: endoplasmic reticulum; FAZ: flagellum attachment zone; glycos.: glycosomal; iso. p.: isoelectric point; isom.: isomerase; methyltransf.: methyltransferase; MICOS: mitochondrial contact site and cristae organisation system; mito.: mitochondrial; MRP: mitochondrial ribosomal protein; mt. SSU: mitochondrial small subunit ribosomal; NOP: nucleolar protein; nucl.: nucleus; nucleol.: nucleolus; prot.: protein; put: putative; r. : ribosomal; RRS1: ribosome biogenesis regulator protein 1; snRNP: small nuclear ribonucleoprotein; struct.: structural; s.u.: subunit; ter.: terminal
