# Supplementary figures and images for: A comparison of three approaches for the discovery of novel tripartite attachment complex proteins in Trypanosoma brucei
Source: PLoS Negl Trop Dis. 2020 Sep 16;14(9):e0008568. doi: 10.1371/journal.pntd.0008568 (PMC7521757; doi:10.1371/journal.pntd.0008568)

**Table S3. TAC102 yeast two-hybrid screen clones.**


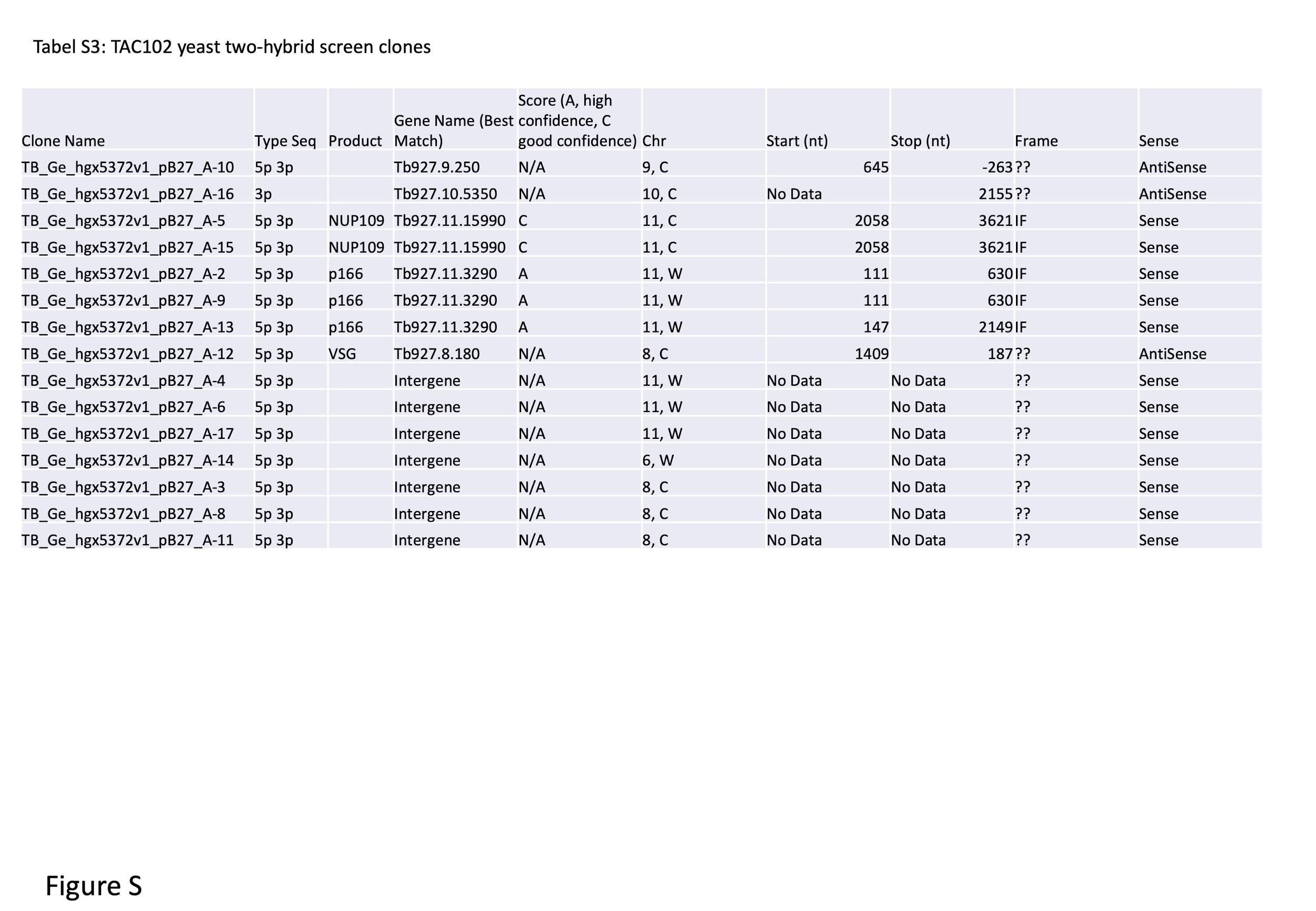

Supplement: S3 Table — (DOCX) [file pntd.0008568.s004.docx]
